# Natural Habitat Design for Zoo-Housed Elasmobranch and Teleost Fish Species Improves Behavioural Repertoire and Space Use in a Visitor Facing Exhibit

**DOI:** 10.3390/ani11102979

**Published:** 2021-10-15

**Authors:** Kristie Lawrence, Sally L. Sherwen, Hannah Larsen

**Affiliations:** 1 Wild Sea, Melbourne Zoo, Melbourne, VIC 3052, Australia; 2 Wildlife Conservation and Science, Zoos Victoria, Melbourne, VIC 3052, Australia; ssherwen@zoo.org.au (S.L.S.); Hannah.Larsen@mpi.govt.nz (H.L.)

**Keywords:** welfare, fish, captive animal behaviour, enclosure design, visitor effect

## Abstract

**Simple Summary:**

Studies investigating whether a captive environment is meeting the needs of the species housed are relatively common among captive mammals. However, studies exploring fish behaviour in captive display enclosures are far less common in the scientific literature. Focusing on a small group of sharks, rays and smaller fish within a single display, our objectives were to; assess whether the behaviours of a select number of individual fishes within a single display are altered after the environment is enriched to enhance environmental complexity and visitor exposure is reduced, and also to increase our understanding of captive fish behaviour to improve capacity for evidence-based management decisions. In summary, increased environmental complexity and reduced visitor interaction showed correlations with increased expression of natural behaviours in all fish studied, including increased space use and decreased abnormal repetitive behaviours in some species. These results reflect a change toward more natural wild behavioural time budgets. Studying behaviour change in fish in different environmental conditions provides a good basis for evidence-based decision making.

**Abstract:**

This study investigated the behaviour of two Elasmobranch species; Southern fiddler ray (*Trygonorrhina dumerilii*, *n* = 1) and Port Jackson shark (*Heterodontus portusjacksoni*, *n* = 4) and two teleost species; moonlighter (*Tilodon sexfasciatus*, *n* = 1) and banded morwong (*Cheilodactylus spectabilis*, *n* = 1) living within a single enclosure. For this study, two treatments were compared, the original enclosure design, and then after the enclosure had been renovated to more closely represent the species natural habitats, with a raised front viewing glass to prevent visitor interaction. Behaviours such as resting, swimming and abnormal behaviours such as surface and perimeter swimming (elasmobranchs only) were recorded as well as location within the enclosure, for 10 days pre and 10 days post renovation. The Port Jackson sharks significantly reduced the performance of abnormal behaviours after renovation, and significantly increased the time spent near the exhibit front. The Southern fiddler ray increased resting post renovation, while the teleost species also spent more time near the exhibit front. Although a small sample size was used, the results suggest that a more naturalistic environment with multiple micro-habitats and effective visitor barriers allows for a greater proportion of the day spent exhibiting natural behaviours, greater space use and reduced stereotypes.

## 1. Introduction

Historically, captive institutions such as zoos and aquariums were guided by a minimum standard when designing animal enclosures, providing perceived adequate environmental conditions for the basic needs for the animal’s survival and physiological health [1,2,3]. These standards were largely based on a limited understanding of the specific and varied behavioural ecologies of the species under care, and perhaps also reflecting the current attitudes towards animal welfare importance of the era [4]. With the zoo and aquarium industry increasingly becoming more welfare-focused, it is now common for enclosures to be designed around the biological functioning and capabilities of the animals, in consideration of potential affective (mental) states/experiences [1,5].

There is now a great deal of targeted research focusing on the relationship between behavioural ecology, environmental complexity and the overall welfare states of captive animals. [1,6,7,8,9]. Using various indicators, including behaviour, numerous studies have emphasised the complexity of an enclosure and the environmental enrichment provided can be critical in facilitating positive welfare outcomes, as well as allowing individuals to express agency through choice and control when confronted with stressors associated with captivity [10,11,12,13,14,15,16]. Enclosures that reflect more naturalistic living conditions have been found to accommodate greater species-specific natural behaviours, likely reflecting more positive experiences in animals [1,10,17,18,19].

Environmental enrichment has been described as a process for improving or enhancing environments aligned with the animal’s behavioural biology and natural history [20]. This can be achieved through varying the stimuli available to the animals across multiple aspects of their day. This variation can result in an increase in complexity within the environment provided, thereby increasing the behavioural choices available to animals, enhancing variation, novelty, choice and control, and has been shown to be a critical aspect to achieving good welfare [10,20,21,22,23,24]. In this study we have focused on physical enrichment and the associated effects on behaviour.

Key behavioural indicators of interest in these animal-welfare-focused studies include behavioural diversity defined as a measure of the richness and frequency of behaviours [25] as well as space use. For example, Chamove et al. [26] found the use of deep wood chip litter scattered in the exhibits of eight primate species led to increased behavioural diversity, with more time spent on the ground foraging and less time engaging in fighting and periods of inactivity. Using instantaneous scan sampling of eight sitatunga, *Tragelaphus spekii*, Rose and Robert [27] found their overall space use was higher in areas of their exhibit that were more biologically relevant and reflected more naturalistic habitat. Abnormal repetitive behaviours can also be a useful behaviour to investigate the impacts of environmental conditions on captive animals [28]. For example, In Southern India, the stereotypic pacing levels of Indian leopards, *Panthera pardus*, in four zoos were found to be significantly lower when on-exhibit in a more enriched space compared to a simpler off-exhibit space [13].

To date, this focus on enclosure impacts on captive animal welfare has largely concentrated on higher class vertebrates and mega fauna [2,5,29,30] and less on species that have vastly differing behavioural repertoires and perceptions to that of humans [31,32]. Studies investigating the behaviour and welfare of fish species housed in zoos and aquariums are extremely limited [33]. Much of the literature addressing fish welfare focuses on questions such as their ability to feel pain, express emotion and whether they are indeed sentient and lead complex lives worthy of a more complex welfare discussion [34,35,36,37]. Numerous studies have now shown that fish do possess similar neuroendocrine and physiological stress responses comparative to higher vertebrates and consciously alter their behaviour in response to noxious stimuli and prolonged periods of stress [8,34,35,36,38]. Beyond the discussion of pain perception, some further research has started to explore fish emotion and cognition [39,40], learning and familiarity [41,42] demonstrating different species’ impressive memory capabilities [16,43], individual recognition abilities and even complex social ranking [44].

Fish welfare indicators that have been studied thus far largely focus on identifying poor welfare states, with limited identification of good welfare indicators [45]. They include; physiological indicators such as plasma cortisol [46], glucose, lactate, performance/growth indicators and behavioural indicators including swimming activity, stereotypies, aggression and surface airbreathing [47]. Changes in feeding behaviour and intake, as well as increased ventilatory activity, have also been used as indicators of poor welfare in fish [45].

Similarly to captive mammals, the knowledge of how different fish species perceive their external environment and how that perception influences behavioural responses and motivation to perform certain behaviours is invaluable for evidence-based decision making [45]. Much of the research in this field has focused on fish species common to the laboratory or farming sector [48] and is demonstrating fish have clear behavioural and physiological changes in response to environmental enrichment and complexity [34,35,49]. For example, zebrafish, *Danio rerio*, showed significantly altered behaviours when housed in varying levels of environmentally enriched tanks [50,51]. Studies of seabream, *Sparus aurata*, showed an increase in space use and exploration with increased environmental enrichment [11], while Alexandre da Silva et al. [43] found *Serrapinnus notomelas*, foraged less when exposed to low levels of enrichment. Zonghang Zhang et al. [16] exposed captive juvenile black rockfish, *Sebastes schlegelii* to differing levels of plant and structure enrichment and found that control fish exposed to no enrichment had significantly higher basal stress levels (defined by cortisol level and opercular beat rate). Additionally, studies of the Nile tilapia, *Oreochromis niloticus*, three-spined stickleback, *Gasterosteus aculeatus* and gold fish, *Carassius auratus*, demonstrate that all of these species exhibit a preference for enriched environments that more accurately mimic their natural habitat [52,53,54,55].

In addition to environmental complexity, another feature of the zoo and aquarium environment that is likely to impact fish behaviour and welfare is exposure to visitors. The visitor effect has been well studied in other taxa, with a range of responses found from negative to positive [56,57,58,59,60,61,62,63]. The proximity to humans combined with the presence or lack of retreat spaces has shown clear behavioural changes in numerous species including intraspecies aggression, vigilance, as well as changes in overall activity levels [58,64,65,66]. The evidence suggests a key determinant of the direction of the response from an animal is likely to be enclosure design and the associated level of control an animal has to manage exposure to visitors [15,58]. However, to date, this has not been well investigated in fish species but is nonetheless a key part of the environment that needs consideration.

Port Jackson shark, *Heterodontus portusjacksoni*, and Southern fiddler ray, *Trygonorrhina dumerilii*, are ideal species for assessing elasmobranch behaviour in captivity. There is limited detailed knowledge of wild elasmobranch behavioural activity and spatial structure [67]; however, it is well known that both species are primarily benthic in nature, meaning they spend a majority of their time on, or near the sea bottom [68,69,70]. Therefore, behaviours at the surface such as swimming breaking the surface water or swimming on their side against the perimeter housing walls, are considered unnatural if frequently observed in captivity. With these surface behaviours undocumented in healthy wild animals, it is predicted that elements of captivity may induce these behaviours and could be reflective of deficiency in their environment. Wild benthic elasmobranch species have also been found to have low vagility [67]. Port Jackson sharks are commonly found resting in caves and gullies in the daylight hours [68] and acoustic monitoring has revealed substantial periods of inactivity overall [69,70,71]. Acoustic monitoring of Southern fiddler rays also found they do not regularly move long distances [72] and are largely encountered on the sea floor resting, or partially submerged under the sand often in dimly lit environments [70,72,73,74]. As non-obligate ventilators, they have well-defined resting and swimming activity periods making these species ideal for captive observations as their activity budgets and space use can be easily documented [71].

Detailed knowledge of the activity budgets of the moonlighter, *Tilodon sexfasciatus* and banded morwong, *Cheilodactylus spectabilis*, the teleost species studied here, is also limited. Wild studies of moonlighter fish have shown some act as cleaner fish within a reef environment, adopting a small home range within caves and crevices where other fish gather for cleaning services, whilst at other times individuals were documented travelling up to 30 m from their shelters cleaning opportunistically [75]. While studies of banded morwongs have not shown any systemic feeding migrations their density has been positively correlated with topographic complexity, and they are known to be diurnally active [76]. Both species, therefore, were considered good subjects due to their wild association with reef structure and complexity [76,77].

This preliminary study aimed to assess the impact of enclosure changes on a selection of fish species. The enclosure changes included; (1) increased environmental complexity through the use of more species-appropriate furnishings and substrate, and (2) reduction of visitor interaction through the addition of a glass barrier preventing visitors from putting their hands into the tank. Ultimately, the aim was to increase our understanding of captive fish behaviour to improve capacity for evidence-based management decisions.

It was hypothesised that after the exhibit is renovated to provide a more naturalistic, safe environment with multiple micro habitats the activity budgets of the elasmobranchs and teleosts will more closely align with wild activity, alongside an increase in space use and an overall reduction in stereotypes.

## 2. Materials and Methods

### 2.1. Study Subjects and General Housing Conditions

Observations were carried out on four Port Jackson sharks (two male and two female), one Southern fiddler ray (female), one banded morwong (sex unknown) and one moonlighter (sex unknown) in a single display tank located at Melbourne Zoo.

The subject animals also shared this tank with 13 Yellow-eyed Mullet (*Aldichetta forsteri*), one Globefish (*Diodon nicthemerus*) two Sea Sweep (*Scorpius aequipinnis*), and one Southern Rock Lobster (*Jasus edwardsii*). Whilst all these species occupy different niches or micro habitats, they are all common to the inshore temperate waters of the Southern Ocean. These animals were not studied as part of this research.

**Figure 1:**
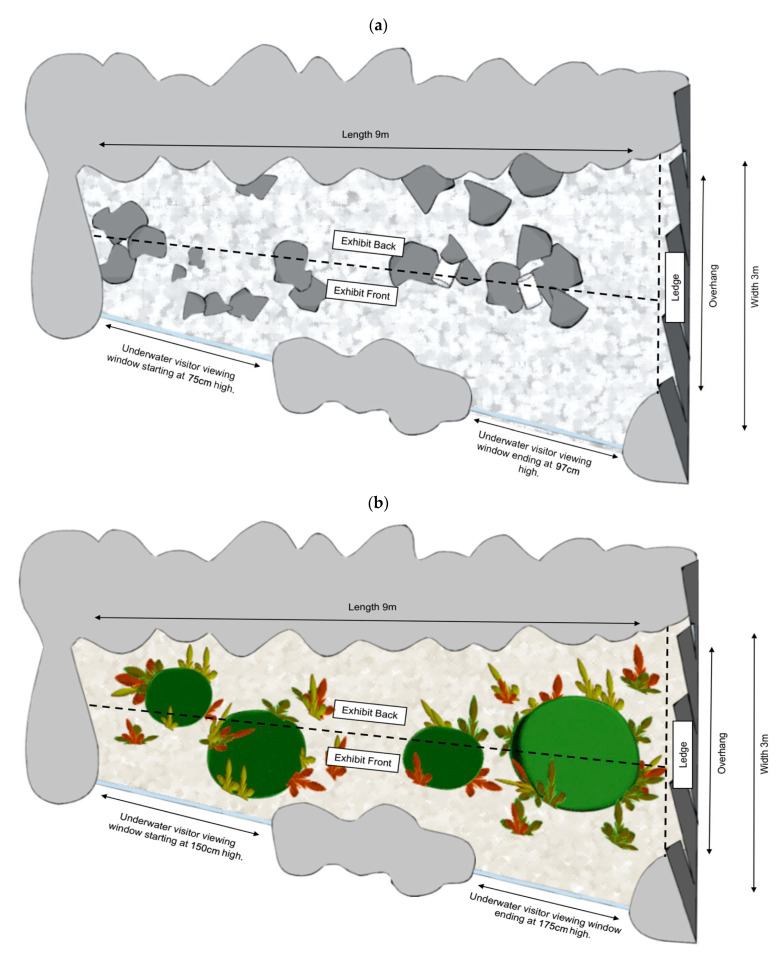
(**a**) The tank at Melbourne Zoo where all species were housed prior to renovations. Dark grey shapes represent large rocky structures (without shelter), two PVC pipes for shelter are represented by white cylinders. (**b**) The tank at Melbourne Zoo where all species were housed post renovations. Dark green and light green shapes represent rocky structures that are textured and provide multiple places for shelter, Multi-colour shapes represent the mock seaweed installed in the exhibit that provides complexity and shelter. The background colour change represents a change in substrate from thick shell grit to fine coral sand. For both (**a**,**b**) light grey areas around the outside of the tank represent mock rock and light blue lines indicate where visitor viewing was possible through glass. Locations for behavioural sampling are indicated by dotted lines and labels.

The tank was located outdoors and under the cover of a shade sail. It measured 8 m long, 3 m wide and the water depth was 1.15 m (Figure 1a). The filtration was designed as a closed filtration system using sand filters, de-nitrifying filters and UV sterilisation. Temperature was controlled via a chiller allowing a minimal seasonal variation of +/−5 degrees and attempting to mimic natural conditions found in the local habitat. The system holds 75,000 L of seawater, 40,000 L within the pool, 18,000 L in the balance tank and 17,000 L within the life support system. The system was topped up post backwashing from raw seawater collected in Port Phillip Bay, Victoria.

### 2.2. Tank Renovations (Treatments)

Fish behaviour and space use was studied under two conditions (Figure 1):

1. Pre-renovation: tank had minimal environmental complexity with very little overhead cover, no seaweed theming, thick shell grit substrate and high visitor exposure through lack of barrier at water’s surface. The height of the glass on the visitor viewing side reached a height of 75 cm at the lowest point, rising to 97 cm at the highest (towards the ledge overhang) as the visitor ground sloped downward. The only structures within the tank included 40/60 cm × 40/60 cm size garden rocks, some clumped together, and two PVC pipes wedged in between two rock clumps providing cover for only small fish.
2. Post-renovation: the tank was renovated to incorporate more naturalistic environmental features including the addition of four themed structures; a large swim through cave, two small enclosed caves with a front entrance and a large bommie structure providing overhead cover, finer coral sand substrate and multiple seaweed clusters. The glass wall was elevated to a height of 150 cm rising to 175 cm at the far side blocking direct visitor contact with water.

The in-tank renovation took 18 days to complete. During this time, animals were housed in two temporary indoor holding tanks, both equivalent size and collective volume of 10,000 L (not inclusive of life support volumes) and on the same filtration system. The Port Jackson sharks and the Southern rock lobster were held in one tank, fiddler rays in the other and the teleost fish equally were split by number between the two.

The tank was visually separated into three locations: exhibit front (1.5 m closest to the visitor viewing area), exhibit back (1.5 m furthest from the visitor viewing area) and a ledge overhang which consisted of the area underneath wall theming that overhangs the tank by 80 cm–1 m running along nearly the full width of the tank on the right-hand side (referred to here on out as the “ledge” location, Figure 1).

### 2.3. Behavioural Observations

An ethogram was developed based on preliminary observations of the individuals over a two-day period before the study began. It was decided to focus on broad state target behaviours for each species (Table 1).

The perimeter, surface swimming and spy hopping are only relevant for the elasmobranchs observed not the teleost fish who were not observed to engage in those behaviours.

Observations were conducted for 10 days in each treatment period: pre-renovation (22 June 2018–11 July 2018) and post-renovation (10 December 2018–10 February 2019). The in-tank renovations occurred in September 2018 and the animals were moved back after completion. The external glass barrier installation occurred in early November and we allowed an additional four weeks post this for animals to settle back into the environment to reduce any potential impact of the temporary housing and glass installation on behaviours.

In addition to recording behaviour, the observers also recorded where each individual was according to the three locations shown in Figure 1. One observer recorded all behaviours and locations from a distance so that there was no interference with normal activity of both study subjects and visitors.

The data were sampled in 6 × 45-min periods between 7 a.m. and 5:45 p.m. A scan sample was conducted every 5 min within the 45-min period and each individual’s behaviour and location was recorded. If spy-hopping behaviour was observed in the elasmobranchs during the observation period it was recorded using an all-occurrence sampling method.

### 2.4. Analysis

All statistical analysis was conducted using JASP Statistical Software (Version 0.14.1 (2020), JASP Team, Amsterdam, The Netherlands). For all scan sampled behaviours and locations, the average proportion of time spent performing each behaviour/time spent in each location was calculated for each individual for each time period. Therefore, there were six units of observation (time periods) per day, per period (*n* = 120 units of observation per individual). For the all-occurrence sampled spy hopping the total count of all occurrences was calculated per day for each treatment (*n* = 20 units of observations per individual). Because there were multiple individuals of the Port Jackson sharks (*n* = 4), their results were analysed as a group, the Southern fiddler ray and fish species were analysed as individuals.

All behaviour and location data were non-normally distributed and could not be transformed. A Wilcoxon’s signed rank test was used to compare each behaviour and location pre-post renovation. W-statistics are presented in the results and *p*-values < 0.05 are considered statistically significant.

## 3. Results

### 3.1. Elasmobranchs

**Figure 2:**
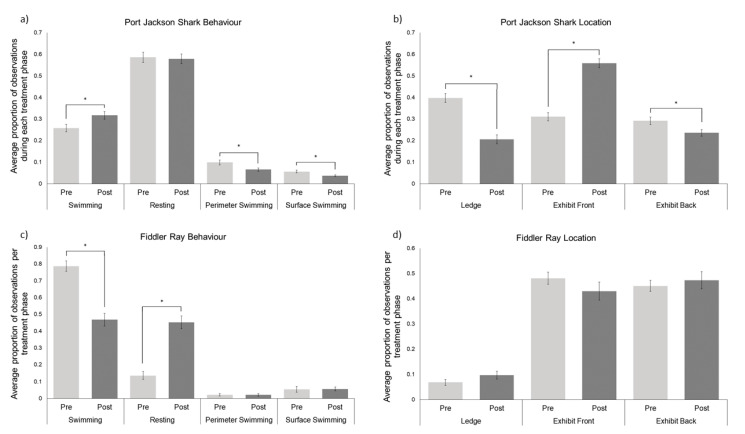
(**a**) The average proportion of time the Port Jackson sharks (*n* = 4) spent performing the different behaviours pre-post renovations. (**b**) The average proportion of time the Port Jackson sharks (*n* = 4) spent in each exhibit location pre-post renovations. (**c**) The average proportion of time the Southern fiddler ray (*n* = 1) spent performing the different behaviours pre-post renovations. (**d**) The average proportion of time the Southern fiddler ray (*n* = 1) spent in each exhibit location pre-post renovations. Pre-renovations means are represented by light grey bars and post-renovations are represented by dark grey bars for all panels. The error bars represent the SEM and the * indicate statistically significant differences (*p* < 0.05).

The average proportion of time spent engaged in swimming behaviour significantly increased for the Port Jackson shark (*n* = 4) from pre-renovation (Mean = 0.26 ± 0.02) to post renovation (Mean = 0.32 ± 0.02; W = 8170.5, *p* = 0.02; Figure 2a). Perimeter swimming decreased from pre-renovation (Mean = 0.10 ± 0.01) to post-renovation (Mean = 0.07 ± 0.01; W = 5879.0, *p* = 0.01), as did surface swimming (pre-Mean = 0.06 ± 0.01, post-Mean = 0.04 ± 0.004; W = 3791.0, *p* = 0.02). There was no difference in resting behaviour pre-post renovation.

Pre-renovation, the Port Jackson sharks spent a greater proportion of time under the ledge (Mean = 0.40 ± 0.02) compared to post-renovation (Mean = 0.21 ± 0.02; W = 15719.0, *p* ≤ 0.001; Figure 2b), and at the back of the exhibit (pre-Mean = 0.29 ± 0.02, post-Mean = 0.24 ± 0.02, W = 10716.0, *p* = 0.03). Whereas the proportion of time spent at the front of the exhibit significantly increased from pre-renovation (Mean = 0.31 ± 0.02) to post-renovation (Mean = 0.56 ± 0.02; W = 5145.5, *p* < 0.001).

There was no statistically significant difference in the daily performance of spy hopping in the Port Jackson sharks from pre-renovation (Mean = 15.17 ± 7.52) to post-renovation (Mean = 11.51 ± 5.67; W = 469.0, *p* = 0.16).

For the Southern fiddler ray (*n* = 1), the average proportion of time spent engaged in swimming behaviour significantly decreased from pre-renovation (Mean = 0.79 ± 0.03) to post-renovation (0.47 ± 0.04; W = 1362.0, *p* = 0.001; Figure 2c). Alternatively, resting behaviour increased from pre-renovation (Mean = 0.14 ± 0.02) to post-renovation (Mean = 0.45 ± 0.04; W = 122.0, *p* ≤ 0.001). There was no difference in the proportion of time spent engaged in perimeter swimming from pre-renovation (Mean = 0.02 ± 0.007) to post-renovation (Mean = 0.02 ± 0.007, W = 59.5, *p* = 1.0), and no difference in the proportion of time spent surface swimming from pre-renovation (Mean = 0.05 ± 0.02) to post-renovation (Mean = 0.06 ± 0.01, W = 177.0, *p* = 0.55). There was no significant difference observed in the proportion of time spent in the exhibit front (pre-Mean = 0.48 ± 0.02, post-Mean = 0.43 ± 0.04, W = 955.5, *p* = 0.20), the exhibit back (pre-Mean = 0.45 ± 0.02, post-Mean = 0.47 ± 0.03, W = 599.5, *p* = 0.55), or at the ledge (pre-Mean = 0.07 ± 0.01, post-Mean = 0.10 ± 0.02, W = 297.5, *p* = 0.13) (Figure 2d).

There was no statistically significant difference in the daily performance of spy hopping in the Southern fiddler ray (*n* = 1), from pre-renovation (Mean = 12.00 ± 4.87) to post-renovation (Mean = 4.30 ± 0.83; W = 36, *p* = 0.12).

### 3.2. Teleost

**Figure 3:**
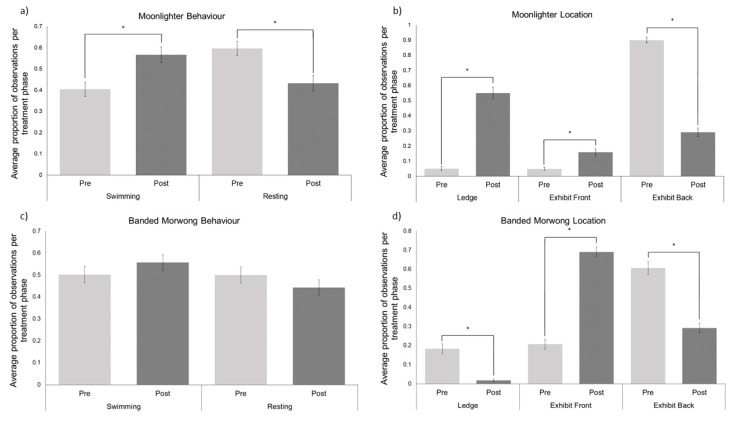
(**a**) The average proportion of time the moonlighter (*n* = 1) spent performing natural behaviours pre-post renovations. (**b**) The average proportion of time the moonlighter (*n* = 1) spent in each exhibit location pre-post renovations. (**c**) The average proportion of time the banded morwong (*n* = 1) spent performing natural behaviours pre-post renovations. (**d**) The average proportion of time the banded morwong (*n* = 1) spent in each exhibit location pre-post renovations. Pre-renovations means are represented by light grey bars and post-renovations are represented by dark grey bars for all panels. The error bars represent the SEM and the * indicate statistically significant differences (*p* < 0.05).

The average proportion of time the moonlighter spent swimming significantly increased from pre-renovation (Mean = 0.40 ± 0.03) to post-renovation (Mean = 0.57 ± 0.04; W = 456.0, *p* = 0.002; Figure 3a). This also reflected in the moonlighter spending significantly less time resting from pre-renovation (Mean = 0.60 ± 0.03) to post-renovation (Mean = 0.43 ± 0.04: W = 1257.5, *p* = 0.002).

The moonlighter spent significantly more time at the exhibit front (Mean = 0.16 ± 0.02) and under the ledge (Mean = 0.55 ± 0.038) post renovation compared to pre-renovation (exhibit front Mean = 0.05 ± 0.01; W = 95.5, *p* ≤ 0.001; ledge Mean = 0.05 ± 0.01; W = 6.0, *p* ≤ 0.001).

There was no difference in the average proportion of time the banded morwong spent engaged in either swimming or resting behaviours pre-post renovation (Figure 3c). The banded morwong spent significantly more time at the back of the exhibit pre-renovation (Mean = 0.61 ± 0.03) compared to post-renovation (Mean = 0.29 ± 0.03; W = 1380.5, *p* ≤ 0.001; Figure 3d). Similarly, the banded morwong spent significant more time under the ledge in the exhibit pre-renovation (Mean = 0.18 ± 0.03) compared to post-renovation (Mean = 0.02 ± 0.01; W = 870.5, *p* ≤ 0.001). Time spent in the front of the exhibit significantly increased from pre-renovation (Mean = 0.21 ± 0.03) to post-renovation (Mean = 0.69 ± 0.03; W = 24.0, *p* ≤ 0.001).

## 4. Discussion

This research has added to the currently limited suite of studies investigating fish behaviour in zoos and aquariums. It provides evidence that enhancing environmental complexity based on species wild habitat can impact the behaviour of benthic elasmobranchs and teleost fish.

### 4.1. Port Jackson Sharks

When environmental complexity was enhanced, Port Jackson sharks increased time spent swimming overall, but decreased time spent perimeter swimming and surface swimming. It is possible that the reductions in these behaviours reflect a more positive experience for the animal as these behaviours are considered abnormal for this species in the wild. Being benthic demersal fish, Port Jackson sharks spend a majority of their time on or very near the sea floor, resting, hunting and swimming [68,69,70,71]. Surface-breaking behaviours are well documented in some species of captive elasmobranchs. In a 2004 study in the UK, “Surface Breaking Behaviour” was documented in nearly three-quarters of public aquaria, making up a third of the stereotypic behaviours studied, predominately exhibited by sharks and rays [78]. These behaviours are thought to be a result of environmental factors, temporal links to feeding schedules and/or methods of feeding are often witnessed in touch pools [79,80,81]. In some captive environments, the frequency of surface-breaking behaviour performed by a group of rays was reduced when provided benthic feeding opportunities [81]. In our case, benthic feeding for the sharks existed in both treatments. Other studies found no correlation with feeding or foraging opportunities, rather, surface-breaking behaviour was more frequent in tanks with less environmental enrichment [80]. Perhaps the significant overall change of habitat to a more species-specific benthos friendly environment, combined with a visitor barrier contributed to the decrease of abnormal repetitive behaviours and surface breaking, leading to a more natural behavioural expression.

Interestingly, we did not see a significant difference in the daily performance of spy hopping in the Port Jackson sharks between treatments. Perhaps this could be explained by the amount of variation in the frequency of spy hopping between individuals, with some performing the behaviour more than others. It is possible that this variation, however, may have been less significant with a larger sample size. Another potential contributing factor not investigated in this study could be the lack of depth variation. Port Jackson sharks are known to migrate from inshore rocky reefs to deeper water at the end of the breeding season (September/October) and may travel up to hundreds of kilometres in migration [82]. Tagged individuals have also shown that they regularly move between different sites within a reef throughout the year [82]. The uniform shallow environment in this enclosure could elicit a behavioural response, such as spy hopping to seek alternate environments and depth.

Resting behaviour remained high and was observed to be the dominant behaviour of the activity budget. This is similar to wild observations where Port Jackson sharks are commonly observed resting and studies have shown large periods of inactivity overall, particularly in daylight hours [68,69,70,71].

Results also showed a change in location in the post-renovation condition. Port Jackson sharks spent less time under the ledge and increased overall space use within the exhibit. In the wild, this species is well-documented to be found resting and spending time in and around caves and gullies, particularly in the daylight hours [68,69]. In pre-renovation conditions, the option for any significant shelter was limited to the ledge space only. Post-renovation conditions provided multiple enhanced features including caves, structures and seaweed clusters across the length of the tank. This allowed the Port Jackson sharks to disperse more when resting and offered more choice over resting space.

More time was also spent at the front of the tank post renovations which could also potentially be attributed to the installation of a glass barrier. Before renovations, the perimeter glass edge was at a lower height, meaning visitors were easily able to reach over the barrier and contact the water and the animals. It is likely this may have been an aversive visitor behaviour that resulted in the Port Jackson sharks avoiding this front section. A similar result was found in a group of captive little penguins, *Eudyptula minor*, who when exposed to visitors, looming over the barrier, showed an increase in avoidance behaviours and increased distance from the visitor viewing area [60].

### 4.2. Southern Fiddler Ray

The Southern fiddler ray showed an increase in resting behaviour, decrease in swimming, but no significant change in time spent in different locations between conditions. The latter can potentially be explained by wild Southern fiddler ray habitat preference. This species is found to spend the majority of their time in open sandy bay areas or seagrass meadows and have not been found to be associated with any structures or significant cover in the wild [68,72,73,74]. The pre- and post-renovation changes did not decrease the open sandy space available throughout the tank, rather substituted the existing low rising rock formations with more complex habitat inspired structures. With relatively the same amount of sand space available, no change was expected. Interesting to note, there was a slight increase in the use of the ledge/overhang space, which between conditions, remained the same in terms of lack of structure. It is possible this was due to a vacation of this area by the Port Jackson sharks allowing the Southern fiddler ray greater opportunity to utilise this sandy patch.

Furthermore, increased environmental complexity and decreased exposure to visitors did not impact the time spent perimeter swimming or surface swimming. These behaviours were already at very low levels (less than 10% of the time budget). Additionally, no significant difference was noted in the performance of spy hopping between treatments. Similarly to the Port Jackson sharks, fiddler rays will often occupy variable depth habitats, up to 100 m [83]; therefore, again, the lack of depth variation could have contributed to the maintenance of this behaviour. This is only one possible factor and clearly, more research, with larger sample sizes, is needed to understand this behaviour and function across both elasmobranch species.

The increase in resting behaviour observed in this species is more aligned with natural activity budgets observed in wild individuals. Benthic elasmobranchs such as the Southern fiddler ray are found to have relatively low vagility in the wild [67] and are often encountered resting in sandy substrate, at times partially submerged [68,72,73,74]. The pre-renovation substrate was a heavy mix of large compacted shell grit, potentially preventing the Southern fiddler rays’ ability to submerge easily and effectively. Post-renovation, this was changed to a finer more natural coral sand that can easily be disturbed. Whilst we did not record this behaviour as a discrete behaviour separate from resting, anecdotally an increase in partial sand submergence was observed. This would be useful to include as a target behaviour for future observational studies.

It is also possible that the increase in resting behaviour was influenced by the reduction in visitor exposure, potentially representing a more relaxed or comfortable state. Other research has suggested similar findings, for example, one study found an increase in resting with reduced visitor numbers, possibly indicative of a relaxed state in a group of captive gorillas [63]. Similarly, studies with captive Koalas, *Phascolarctos cinereus*, and Sulawesi crested macaques, *Macaca nigra*, found that there was an increase in visitor-related vigilance and decrease in resting states with increasing visitor numbers [57,59].

### 4.3. Moonlighter and Banded Morwong

The moonlighter significantly increased swimming behaviour and decreased resting behaviour in the post-renovation condition. In the wild, this species commonly shelters in shallow water caves, crevices and jetty’s with complex reef habitats but studies have shown they can move great distances within this habitat to forage [75]. The previously dominant resting behaviour was shown to be mainly exhibited in the back area of the tank. Whilst this space previously had no real significant shelter, a planted garden pocket behind the tank did provide some overhead shadowing, as branches stretched out over the water. In post-renovation conditions, multiple microhabitats and shelters were now distributed throughout the length of the tank, providing safe movement. Also, interesting to note, in post-renovation conditions, not only was there an increase in swimming time but also an increase in time spent under the ledge/overhang area. This correlates with the Port Jackson sharks decreasing their time spent in this space. It is possible that with fewer sharks resting in the area, the moonlighter felt more comfortable utilizing this shelter. These inter-species interactions are likely to be a factor that influences individual animal behaviour and warrant further investigation.

The banded morwong is also a shallow demersal reef fish and can be found in areas with greater topographic complexity [76,84]. As such, with increased tank complexity we expected to see some changes in behaviour akin to the moonlighter. Whilst the banded morwong activity did not significantly differ between treatments, both fish did significantly increase their time spent at the front of the tank and decreased time spent at the back of the tank. This is similar to what was observed in the Port Jackson sharks. It is possible that this reduced avoidance behaviour once the potentially aversive stimuli (visitor exposure and risk of hands in tank) was removed supporting the visitor effect hypothesis.

## 5. Limitations and Future Research

This study was designed as a preliminary investigation into environmental change effects on target species of elasmobranchs and teleosts. The small sample size of individuals limits our ability to extrapolate results to individuals in other situations. Nonetheless, it is common for these small-scale interventions to occur in zoos and aquariums, and it is worth conducting such studies to better understand the impact of these changes to support evidence-based management [85,86,87,88]. It would be of value for other studies to also investigate the impact of enclosure changes in other species, using comparative approaches to facilitate collective learning and insight into the importance of ecological factors on species, ultimately leading to better-designed environments [87,88].

This study focussed on better understanding the impact of enclosure changes on target behaviours including space use and swimming activity, as such, the ethogram was not extensive and we are limited in our ability to interpret the effect of environmental changes on other behaviours. Future work should be directed towards a more detailed investigation of behavioural changes in these species such as aggression, feeding, play, social interactions, breeding behaviours and territorial interactions. Furthermore, a more in-depth assessment of enclosure use would be interesting to explore using space use indices to provide more detail on how the animals utilise the different areas of the enclosure

It should also be noted that this study was conducted in a mixed-species exhibit. The focus was on the elasmobranch species and a select number of teleost fish that are easily observed and we did not explore interactions between these species and other species housed in this environment. Previous studies have found that environmental modifications and enrichment can decrease aggressive interactions and increase the diversity and frequency of affiliative interactions between naturally associating species [89], with some research even identifying interspecies commensalism occurrences [90].

Moreover, future research should cover a 24-h period due to the crepuscular nature of some of the study species. It would be interesting to assess the nocturnal activity budgets of the elasmobranchs to see whether their activity increases analogous to their wild twilight foraging behaviours as seen in research by Kadar et al. [71]. As keepers do not provide food overnight, it would be very interesting to see if this wild-type natural behaviour still persists. Conversely, it would also be interesting to note whether the teleost fish decrease their swimming activity and space use, remaining more sedentary at night time akin to wild type predator avoidance behaviour.

Future experimental work should also tease apart and differentiate between the environmental effects vs visitor effects. A deeper understanding of visitor effects in these settings would be of importance to many captive display facilities given the commonality of housing elasmobranchs in “touch tanks”, and potential welfare implications. Moreover, to determine if these behavioural changes are reflective of underlying welfare changes in these species, it would be of value to explore a greater suite of welfare indices. The assessment of stress hormone concentration has been used in previous studies on other species [91,92,93,94,95,96] however, as far as the authors are aware, the non-invasive viable assessment of cortisol concentration is yet to be fully validated for these species [46]. This would be of significant value for future research to support better welfare outcomes for species housed in these settings. Furthermore, a greater understanding of natural wild time budgets is needed for all such species housed in captivity as this research has demonstrated the value of species-specific enclosure design to facilitate more natural behavioural opportunities.

## 6. Conclusions

In summary, increased environmental complexity and the additional visitor barrier resulted in increased expression of natural behaviours in all fish studied. The Port Jackson sharks increased space utilisation and decreased abnormal behaviours, while the Southern fiddler ray increased natural resting behaviours. Both teleost species, the moonlighter and the banded morwong, increased space use with the moonlighter also showing an increase in time spent swimming. These results are more closely aligned with wild behavioural activities for these species. Studying the behaviour change of fish in different environmental conditions provides a good basis for evidence-based decision making and highlights the value of more species-specific environmental design.

## Figures and Tables

**Table 1:** Behavioural ethogram used for elasmobranch and teleost species in tank pre-post renovations. The sampling method indicates whether the behaviour was sampled using the instantaneous scan sampling method every five minutes during the observation period or using the all-occurrences method throughout the observation period.

Behaviour Type/Label	Description	Sampling Method
Active	Swimming below the water surface at a consistent/steady pace throughout the tank (not in a repetitive pattern).	Scan
Resting	Being motionless on the sand or structure, at rest	Scan
Perimeter Swimming	Moving against the perimeter of the tank in a horizontal position, meaning the ventral surface of the animal is in contact with the glass/tank walls.	Scan
Surface Swimming	Moving with a portion of body out of the water	Scan
Spy Hopping	Vigorously propelling the body out of the water in a vertical motion. At minimum, the head breaches the water but can extend to half the body length. Typically occurs at the edges of the tank.	All-occurrence

## Data Availability

The data presented in this study are available on request from the corresponding author. The data are not publicly available due to organisation policy.
